# Partial sacrectomy with en bloc tumor resection without instrumentation. What level is safe?

**DOI:** 10.1016/j.bas.2025.104246

**Published:** 2025-03-27

**Authors:** Jan Štulík, Michaela Rybárová, Pavel Hladík, Robert Lischke, Zdeněk Klézl, Radek Kaiser, Ondřej Naňka

**Affiliations:** a Center for Spinal Surgery, 1st Faculty of Medicine, Charles University and University Hospital Motol, Prague, Czech Republic; b 1st Faculty of Medicine, Charles University, Prague, Czech Republic; c Institute of Anatomy, 1st Faculty of Medicine, Charles University, Prague, Czech Republic; d III. Department of Surgery, 1st Faculty of Medicine, Charles University and University Hospital Motol, Prague, Czech Republic; e Department of Spinal Surgery, Oxford University Hospitals NHS Foundation Trust, Oxford, UK; f Department of Anatomy, Second Faculty of Medicine, Charles University, Prague, Czech Republic

**Keywords:** Sacrectomy, en bloc sacrectomy, en bloc resection, Sacral tumor, Primary sacral tumor, Chordoma

## Abstract

**Introduction:**

*En bloc* sacrectomy is an extensive surgical procedure which is often the only option which provides cure. Our experience shows that, in selected cases, instrumentation is not necessary even in case of a high *en bloc* sacrectomy retaining the cranial part of the sacrum *in situ*. This creates suitable conditions for subsequent proton therapy.

**Research question:**

What level of resection is safe without reconstruction?

**Material and methods:**

Between 2014 and 2023 we performed a total of 29 sacral resections for various etiologies. Patients following reconstruction of the lumbosacral region by internal fixator (3) and patient after hemicorporectomy (1) were excluded from the study. The study group comprised 25 patients, 15 men and 10 women with a mean age of 45.1 years (range, 1.7–72.2 years). The most frequent indication for surgery was chordoma (8), followed by MPNST (4), yolk sac tumor (2) and undifferentiated sarcoma (2).

**Results:**

Stress fractures of the sacral stump occur in elderly patients with lower bone mineral density, or in younger patients with a higher bone mineral density who are more active when resuming their daily routine after the operation.

**Discussion and conclusion:**

Instrumentation is, in our view, primarily indicated in younger and more active patients, whereas in most cases, even with lower bone mineral density, non-instrumented procedure results in sufficient stability in all levels of partial resection.

## Highlights

• *En bloc* sacrectomy is an extensive surgical procedure which is often the only option which provides cure.
• In selected cases, instrumentation is not necessary in case of a high *en bloc* sacrectomy retaining the cranial part of the sacrum, because it creates suitable conditions for subsequent proton therapy.
• Instrumentation is primarily indicated in younger and active patients, whereas in a majority of patients, *even* with lower bone mineral density, non-instrumented procedure results in sufficient stability.

## Authors’ contributions

Jan Štulík and Zdeněk Klézl contributed to the study conception and design. Michaela Rybárová; collected radiographic and clinical data. All authors contributed to the analysis and interpretation of acquired data. Jan Štulík obtained administrative and technical support necessary for successful completion of the project. Jan Štulík and Michaela Rybárová; were responsible for drafting of the first version of the manuscript. Finally, all authors read, reviewed and approved the final version of the manuscript.

## 1 Introduction

Sacral tumors present one of the most challenging pathologies in spinal surgery. Primary sacral tumors are rare, diagnosed mostly at an advanced stage, with a large extraosseous component. The intricate anatomy of the pre-sacral space and the neural structures inside sacrum complicate surgical treatment (Fourney et al., 2005; Guo et al., 2013; Hsieh et al., 2009; Hugate et al., 2006). *En bloc* sacrectomy is often the only option for patients to be cured (Feghali et al., 2021; Fourney et al., 2005). Extensive resections pose a problem of biomechanical instability of the pelvic ring and transmission of forces from the body to the lower extremities. Functional impairment after resection of neural structures significantly affects the postoperative condition. Achieving continuity between the spine and the pelvic ring and prevention of instability is the goal of reconstruction techniques, often with the use of internal fixators, mesh cages, 3D printed anatomical implants, or their combination (Morales-Codina and Martín-Benlloch, 2022; Murakami et al., 2002; Wei et al., 2019; Yu et al., 2010). In contrast, instrumentation may not be necessary in cases of a high *en bloc* partial sacrectomy retaining the cranial part of the sacrum *in situ*, in order to create suitable field for subsequent proton therapy. However, the question is whether the retained part of the sacrum can provide adequate stability in the future. The aim of the study was to evaluate the pelvic ring stability after partial *en bloc* resection of the sacrum and determine a safe level of resection not requiring instrumentation.

## 5 Methods

### 5.1 Patients

Between 2014 and 2023 we performed a total of 29 sacral resections for various pathologies. Patients following reconstruction of the lumbosacral region by internal fixator (3) and patients after hemicorporectomy (1) were excluded from the study. Only patients with *en bloc* partial sacral resection without instrumentation were included. The study protocol was approved by the ethical committee of our hospital. All patients provided written consent to be enrolled in the study and allowed publication of data. All patients were evaluated in terms of surgical parameters, oncological parameters, clinical and functional results, radiological results including bone quality, and complications of surgical and oncological treatment. The study group comprised 25 patients, 15 men and 10 women with a mean age of 45.1 years (range, 1.7–72.2 years). The most frequent indication for surgery was chordoma in 8 cases, followed by malignant peripheral nerve sheath tumor – MPNST (4), yolk sac tumor (2) and undifferentiated sarcoma (2). The main clinical symptoms included coccygodynia radiating into the buttocks and lower extremities, radiculopathy similar as in lumbar disc herniation, urinating or defecating difficulties, or non-specific sensation of pressure in the region of the small pelvis.

### 5.2 Preoperative protocol

All patients underwent complete preoperative testing, including radiographic, CT, MRI and angiographic examinations. Preoperatively, open biopsy was performed in 22 patients and fine-needle aspiration biopsy in 3 cases (11 patients underwent the procedure at our department and 14 patients at another institution). We did not perform preoperative embolization of the regional blood vessels. Radiological examination revealed a tumor involving S2-S5 in 14 cases, S3-S5 in 3 cases, S4-S5 in 2 cases, and unilateral involvement of S1-S2 in 6 cases. Tumor extent was evaluated according to the Enneking (Enneking et al., 1980), Weinstein-Boriani-Biagini (Boriani et al., 1997) and Fourney classifications (Fourney et al., 2005) (Table 1). Four patients received preoperative neoadjuvant chemotherapy/radiotherapy, including one patient treated at our department and three patients referred from other departments where they underwent neoadjuvant chemotherapy/radiotherapy and operation). Patients with chordoma received postoperative proton beam therapy and those with osteosarcoma adjuvant chemotherapy.

**Table 1:** **–** Summary of diagnostic, pre– and postoperative clinical data stratified by type of resection.

Pt	Sex	Age	Diagnosis	Enneking^a^	WBB^b^	Fourney^c^	Resection level	Sacrificed roots	Resection margin	FUP (m)	Recurrence and/or meta (m)	Final disease status
1	F	51,7	osteosarcoma	IIB	A-C/12-9	midline IV	S1-2 (high)	S1+ (S2-5)	wide	117		NED
2	F	14,8	MPNST	IIB	A-E/1-12	midline III	S1-2 (high)	S1+ (S2-5)	marginal	11	local (9)	DOD
3	M	36,6	MPNST	IIB	A-C/2-8	midline III	S1-2 (high)	S1+ (S2-5)	wide	100		NED
4	M	40,2	MPNST	IB	A-C/1-3	midline III	S1-2 (high)	S1+ (S2-5)	marginal	6	lung (4)	DOD
5	F	42,0	HG sarcoma	IIB	A-E/1-12	midline III	S1-2 (high)	S1+ (S2-5)	marginal	3	widespread (1)	DOD
6	M	40,9	chordoma	IIB	A-E/1-12	midline III	S1-2 (high)	S1+ (S2-5)	wide	18	lung (15)	DOD
7	F	63,0	MF histiocytoma	IIB	A-C/12-5	midline IV	S1-2 (high)	S1+ (S2-5)	wide	27	local (24)	DOD
8	M	48,0	chordoma	IIB	A-E/1-12	midline III	S1-2 (high)	S1+ (S2-5)	wide	54	lung (50)	DOD
9	F	59,3	MPNST	IIB	A-C/1-12	midline III	S1-2 (high)	S1+ (S2-5)	wide	57		NED
10	M	72,2	ccRCC	IIB	A-C/7-1	midline III	S1-2 (high)	S1+ (S2-5)	wide	17	widespread (14)	DOD
11	F	32,0	chordoma	IIB	A-E/1-12	midline IV	S1-2 (high)	S1+ (S2-5)	wide	39	lung (30)	DOD
12	F	49,7	chordoma	IIA	B-C/3-10	midline III	S1-2 (high)	S1+ (S2-5)	wide	51	local (33)	AWD
13	F	66,4	chordoma	IIB	A-E/1-12	midline IV	S1-2 (high)	S1+ (S2-5)	wide	21		AWD
14	M	51,5	chordoma	IIB	A-E/1-12	midline IV	S1-2 (high)	S1+ (S2-5)	wide	12		AWD

**Average:**		**47,7**								**38,1**		

15	M	64,2	chordoma	IIB	A-E/1-12	midline II	S2-3 (middle)	S2+ (S3-5)	wide	35	local (20)	AWD
16	M	3,1	yolk sac	IIB	A-C/2-8	midline II	S2-3 (middle)	S2+ (S3-5)	wide	9	widespread (6)	DOD
17	F	71,8	adenocarcinoma	IB	A-C/5-11	midline II	S3-4 (middle)	S3+ (S4-5)	wide	33	widespread (26)	DOD
18	M	1,7	yolk sac	IB	A-B/8-11	midline I	S4-5 (low)	S3+ (S5)	wide	6		NED
19	M	57,1	chordoma	IIB	A-C/2-6	midline I	S4-5 (low)	S3+ (S5)	wide	15		NED

**Average:**		**39,6**								**19,6**		

20	M	37,7	benign cyst	IIB	A-C/7-1	eccentric C	left unilateral	left S3-5	wide	101		NED
21	M	71,8	HCC	IB	A-C/2-5	eccentric B	right unilateral	right S1-2	wide	18	widespread (14)	DOD
22	M	48,3	leiomyosarcoma	IB	B-C/10	eccentric B	left unilateral	left S2-3	wide	50	widespread (45)	DOD
23	M	17,6	UD sarcoma	IB	B-C/9-11	eccentric C	left unilateral	left S2-3	wide	45	local (33)	DOD
24	F	26,8	chondrosarcoma	IB	A-C/2-4	eccentric B	right unilateral	0	wide	27		NED
25	M	13,0	osteoid osteoma	II	B/2-3	eccentric A	right unilateral	0	wide	6		NED

**Average:**		**43,0**								**42,2**		

*Abbreviations:***AWD** = alive with disease, **ccRCC** = clear cell renal cell carcinoma, **DOD** = dead of disease, **HCC** = hepatocellular carcinoma, **HG** = high grade, **MF** = malignant.

fibrous, **MPNST** = malignant peripheral neural sheath tumor, **NED** = no evidence of disease, **UD** = undifferentiated, **WBB** = Weinstein-Boriani-Biagini.

a Enneking et al., 1980

b Weinstein-Boriani-Biagini 1976

c Fourney et al., 2005

### 5.3 Surgical technique

**Fig. 1:**
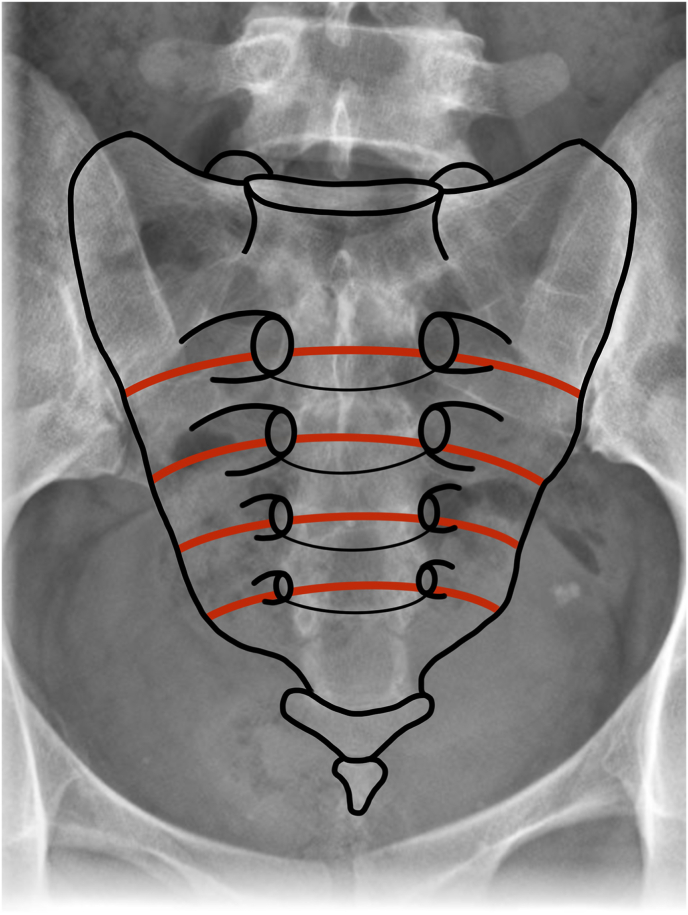
Schematic drawing of respective levels of performed osteotomies, most often S1-S2 interval at the level between S1 foramen or more caudally.

**Fig. 2:**
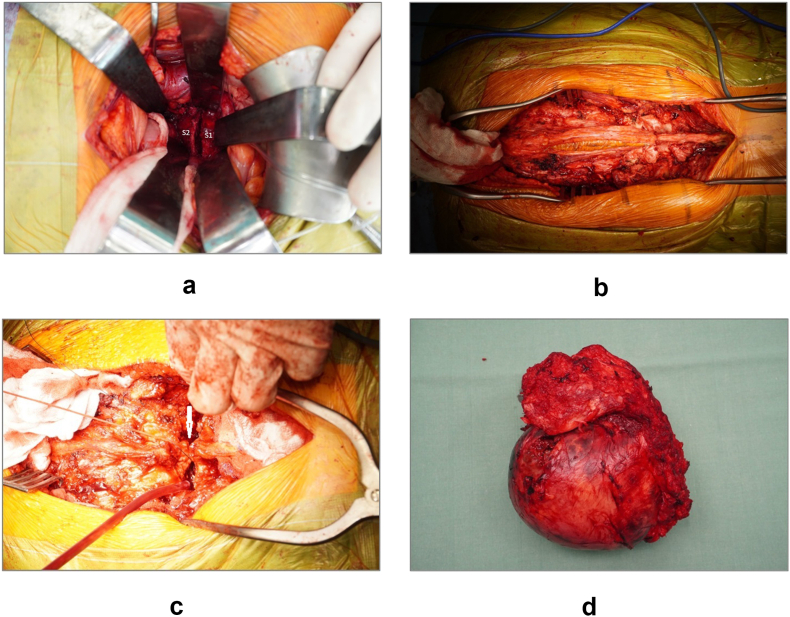
Peroperative photographs of a one stage anteroposterior approach. Anterior osteotomy at the level between S1 foramen (a), the biopsy channel/the original scar including the skin was left on the resected part (b), the dural sac with roots ligated (c, white arrow), the surrounding ligaments and nerve roots were cut and the resected part with the tumor was removed *en bloc* (d).

**Fig. 3:**
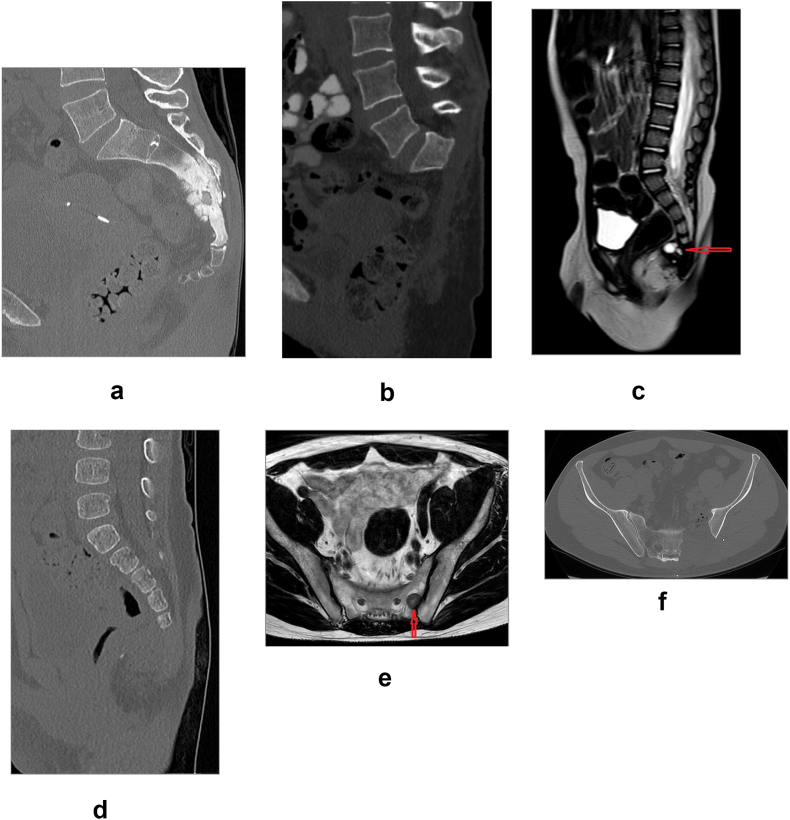
Examples of different levels of resection. CT reconstructions in sagittal plane of high level resection (S1-S2) for osteosarcoma in 51 + 7 female patient pre- (a) and postoperatively (b). 1 + 4 male patient with yolk sac tumor (red arrow) preoperative MRI T2-weighed sagittal image (c) and CT reconstruction in sagittal plane after low level resection (S4-S5) (d). 48 + 3 male patient with leiomyosarcoma metastasis (red arrow) in T2-weighed axial cut of MRI (e) after unilateral resection in axial cut of CT reconstruction (f).

Patients in need of high-level resection (S1-S2) underwent terminal sigmoideostomy three weeks prior to the actual sacrectomy for stool derivation and maintenance of hygiene in the anal area. The operative procedure consisted of one stage anteroposterior approach or one stage posterior approach. In the first stage, the patient was in supine position, a lower midline laparotomy was performed first, for transperitoneal approach exposing internal iliac arteries on both sides, which were ligated, same as internal iliac veins. Subsequently, osteotomy was performed in the respective level, most often S1-S2 interval at the level between S1 foramen or more caudally (Fig. 1). In the next step, a silastic sheet was inserted, the peritoneum sutured, and the surgical wound closed in layers without drain. The patient was then turned to prone position. The posterior approach consisted of incision in the midline at the L4-L5 level, caudally as far as the beginning of the gluteal cleft, where it was turned to the left or right above the buttocks, depending on tumor localization. In case of open biopsy or a previous procedure, the biopsy channel/the original scar including the skin was left on the resected part. Paraspinal, parasacral and gluteal muscles were released to visualize the posterior surface of the sacrum and the coccyx, both sacroiliac joints and iliac crests. The spinal canal was accessed at the respective level according to the type of resection (most often at the L5-S1 level), the dural sac with roots were ligated (usually S2 and caudally). Subsequently, osteotomy was performed in the middle part of the sacrum depending on tumor localization (usually between S1 foramina) and widened into the anterior osteotomy. We took particular care to ensure a safe osteotomy of the lateral part of the sacrum toward the sacroiliac joint. We never use a high-speed burr during *en bloc* tumor resection to eliminate the risk of tissue micro-dissemination and prefer using various types of osteotomes instead. Next, osteotomy was performed of both iliac crests and sacroiliac joints were released. The surrounding ligaments and nerve roots were cut and the resected part with the tumor was removed *en bloc* (Fig. 2). The silastic sheet was then extracted, and paravertebral muscles were reconstructed. Neither muscle flap nor skin transfer was used. Fascia, subcutaneous tissue and skin were closed in layers and subfascial suction drain was inserted. Stabilization by an internal fixator was not used in the study group, in order to create optimal conditions for proton beam therapy. For examplesof different levels of resection see Fig. 3.

### 5.4 Postoperative protocol

Depending on the type of neurological lesion, patients were then placed to the spinal or rehabilitation unit to practice new defecation and urination stereotypes and gait. Postoperatively, patients were mobilized with the support of crutches or a high walking frame and recommended to use them for 3 months. Postoperatively, radiographs and CT scans were performed to check the extent of resection. Biopsy was assessed for quality of margins of the resection. Regular follow-ups were performed at 6 weeks, 12 weeks, 6 months, 9 months, 12 months and then as needed, however, minimally once in 6 months, included radiographic examination; CT and MRI examination at 6 months and 12 months. After one year, follow-ups were scheduled according to the need and development of the oncological condition.

## 3 Results

The study group of 25 patients was divided into 3 groups: 1. high level (S1+), 2. low level (S2+ to S4+) and 3. unilateral (S1-S5). The mean follow-up was 33.3 months (range 3–117 months). The basic demographic characteristics are summarized in Table 1.

### 3.1 Surgical results

The first group (anterior + posterior procedure) comprised 14 patients, the mean overall operative time was 334.7 min (range 240–540min); the mean overall blood loss was 2850 ml (range 5–5400 ml) and the mean postoperative length of stay (LOS) was 37.3 days (range 8–64 days). The second group (posterior approach only with one patient having combined approach) included 5 patients, the mean operative time was 169 min (range 15–270min); the mean blood loss was 411 ml (range, 5–1100 ml) and the mean postoperative LOS was 39.4 days (range, 10–84 days). The third group (posterior procedure only) included 6 patients; the mean operative time was 165 min (range 50–360min); the mean blood loss was 1664 ml (range 50–8000 ml). The mean postoperative LOS was 15.6 days (range 6–46 days). For further details see Table 2.

**Table 2:** **–** Summary of surgical and radiological data stratified by type of resection.

Pt	Sex	Age	Diagnosis	Bone resection level	Surgical time A/P (min)	Blood loss A/P (ml)	X-ray time A/P (s)	Surgical stages	LOS (d)	S1 BMD^a^ (HU)	stress fr. Denis	postop. complications
1	F	51,7	osteosarcoma	S1-2 (high)	120/200	200/1300	2/18	A + P	13	236	zone 1 + 3	
2	F	14,8	MPNST	S1-2 (high)	150/240	100/1000	2/10	A + P	39	260		
3		36,6	MPNST	S1-2 (high)	100/360/50	300/3100/100	4/10/0	A + P + A	16	255		
4	M	40,2	MPNST	S1-2 (high)	110/300	100/1800	4/34	A + P	8	243		wound revision
5	F	42,0	HG sarcoma	S1-2 (high)	120/390	200/2400	2/7	A + P	31	233		
6	M	40,9	chordoma	S1-2 (high)	140/400	300/3200	3/21	A + P	38	207	zone 3	wound revision
7	F	63,0	MF histiocytoma	S1-2 (high)	90/210	200/1600	3/9	A + P	57	302		
8	M	48,0	chordoma	S1-2 (high)	150/390	300/4200	1/5	A + P	16	290		
9	F	59,3	MPNST	S1-2 (high)	210/150	300/2000	2/10	A + P	42	186	zone 3	
10	M	72,2	ccRCC	S1-2 (high)	210/200	400/5000	1/10	A + P	64	183	zone 1	
11	F	32,0	chordoma	S1-2 (high)	120/330	200/2000	3/2	A + P	60	249	zone 3	wound revision
12	F	49,7	chordoma	S1-2 (high)	90/180	50/1600	4/20	A + P	63	354		wound revision
13	F	66,4	chordoma	S1-2 (high)	90/150	300/600	1/2	A + P	28	187		
14	M	51,5	chordoma	S1-2 (high)	100/265	200/4500	4/12	A + P	47	313		wound necrosis

**Average:**	**47,7**				**128,6/243,2/50**	**226/2450/100**	**2,6/12,1/0**		**37,3**	**249,9**		

15	M	64,2	chordoma	S2-3 (middle)	90/180	100/1000	10/2	A + P	84	236		ileus
16	M	3,1	yolk sac	S2-3 (middle)	180	150	10	P	21	352		
17	F	71,8	adenocarcinoma	S3-4 (middle)	180	500	5	P	32	144		
18	M	1,7	yolk sac	S4-5 (low)	15	5	6	P	50	244		
19	M	57,1	chordoma	S4-5 (low)	200	300	10	P	10	260		

**A verage:**	**39,6**				**90/151**	**100/391**	**10/6,6**		**39,4**	**247,2**		

20	M	37,7	benign cyst	left unilateral	75	400	2	P	7	352		
21	M	71,8	HCC	right unilateral	360	8000	11	P	25	193		
22	M	48,3	leiomyosarcoma	left unilateral	200	1000	30	P	8	297		
23	M	17,6	UD sarcoma	left unilateral	200 + 120	1300/50	26/2	P + P	46/8	302		
24	F	26,8	chondrosarcoma	right unilateral	150	800	32	P	6	272		
25	M	13,0	osteoid osteoma	right unilateral	50	100	8	P	9	176		

**Average:**	**43,0**				**165**	**1664**	**15.9**		**15,6**	**265,3**		


*Abbreviations:***A =** anterior approach, **BMD** = bone mineral density, **LOS** = length of stay**, HU** = Hounsfield unit, **P =** posterior approach.

^2^**Denis** et al., 1980

a **Schrieber** et al., 2014

### 3.2 Oncological results

In the first group, a wide surgical margin was confirmed in 11 patients, marginal in 3 patients and there was no contaminated margin. In the second group, wide resection was found in all patients. In the third group, wide surgical margin was found in 4 patients, marginal in 2 patients, again, no contaminated margin was found. Local recurrence was later found in 6 cases. Patient survival times are included in Table 1, Kaplan-Meyer estimate of patient survival is shown in graph 1.

### 3.3 Clinical and functional outcome

We preferred to perform terminal sigmoideostomy in patients who underwent high resection (S1-S2) and in older patients with middle or low resection (S3+), which proved useful in practicing self-care after the operation and improved the standard of hygiene within the surgical wound site. All patients were placed in the spinal unit after stabilization to practice gait and self-care associated with sphincter dysfunction (fecal/urinary incontinence, or necessity of manual evacuation of stool and clean intermittent self-catheterization for the management of urinary retention). A total of 21 patients were able to walk early with crutches or in a high walking frame and gradually without any support. In the first group, plantar flexion of the foot was preserved in all patients. Five patients sustained a stress fracture of the sacral stump which, however, was clinically asymptomatic; the most serious complication was neuropathic pain. The patient's mobility was virtually unrestricted. Stability of the lumbo-sacroiliac complex was not impaired.

### 3.4 Radiological results

**Fig. 4:**
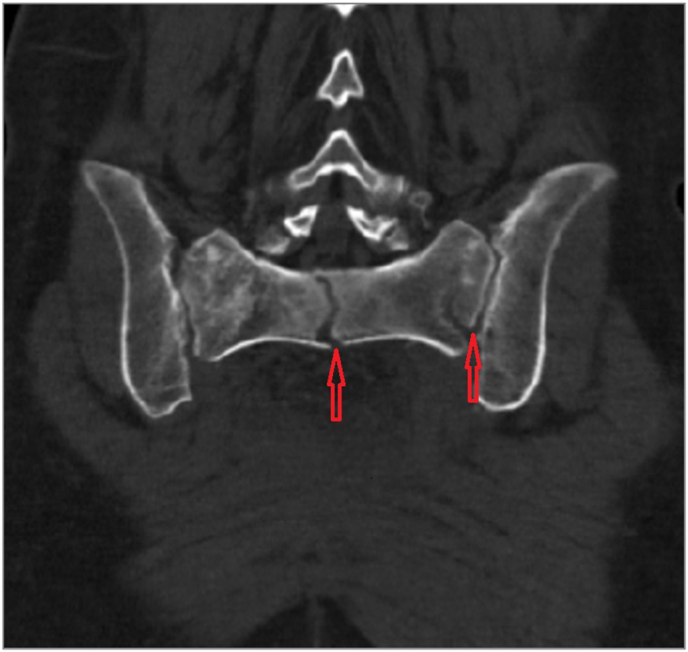
55 + 7 female patient 4 years after high level resection (S1-S2) for osteosarcoma with fractures (red arrows) of the residual part of the sacrum (Denis zones I + III), CT reconstruction in a coronal plane.

In the first group, we identified 5 fractures of the residual part of the sacrum (three in Denis zone III, one in Denis zone I and one in Denis zones I + III), see Fig. 4. The mean HU (Hounsfield unit) value of S1 body in patients with fractures was 212.2 (range, 183–249) and in patients without a fracture 263.9 (range, 187–354). In all of the cases, the values corresponded with the normal bone mineral density (Schreiber et al., 2014). In younger patients, trabecular remodeling of the residual sacrum and sclerotization of the SI joint occurred within 3 months, in elderly patients the remodeling process was slower. Bone quality in patients after a high S1+ sacrectomy was usually slightly lower in patients with a fracture of the sacral stump (S1) as compared those without a fracture (Table 2). The age of the patients did not seem to play a role – the youngest of the patients with a fracture was 32 and the oldest 72.2 years old; the youngest of the patients without a fracture was 14.8 and the oldest 71.8 years old.

### 3.5 Complications

Intraoperatively, we encountered one case of extensive bleeding from post-radiation altered veins during the anterior approach, which was addressed by ligation in cooperation with a vascular surgeon. No other intraoperative complications were observed. Early postoperative complications included wound healing problems in 6 patients, 5 required surgical revision and 1 patient underwent surgical revision of the abdominal cavity for postoperative ileus. (Table 2). In addition to the above-mentioned fractures, late complications included 2 cases of wound dehiscence after the proton beam therapy (70Gy). The therapy was in both cases difficult and protracted, the soft tissue defect failed to heal, and the patients lived with chronic fistula.

## 4 Discussion

Resection of sacral tumors is utilizing extensive and complex operative techniques and is associated with longer operative times, higher blood loss, surgical wound healing issues and, in general, with a higher number of various complications (Varga and Lazary, 2010). The main issues in this respect include the choice of an adequate surgical approach, assessment of stability of the lumbo-sacroiliac complex, filling the space after sacral resection, postoperative wound care and managing of functional disorders following resection of neural structures (Feghali et al., 2021; Fourney et al., 2005; Varga and Lazary, 2010).

Fourney et al. (2005) categorized sacral resections into two basic groups: midline tumors and eccentric lesions. The midline group includes low, middle and high resection, total sacrectomy and hemicorporectomy. The resection level was specified according to the nerve root sacrifice. Low resection is associated with sacrificing S4 and lower nerve roots, middle resection is at the level of S3 nerve roots and in high resection at least one S2 nerve root and both S1 roots. Total sacrectomy requires sacrifice of both S1 roots. In the case of hemicorporectomy (translumbar amputation), neural structures are ligated at the L4-L5 or L3-L4 level (Štulík et al., 2020). The lateral group includes resection of the sacroiliac joint and hemisacrectomy. Total *en bloc* sacrectomy and high sacrectomy are traditionally performed via the anteroposterior combined approach, the middle and low resections via the posterior approach and the lateral resections from the lateral approach (Fourney et al., 2005; Varga et al., 2014; Zhang et al., 2003). Clarke et al. (2012) recommend the use of the posterior-only approach for all sacral tumors that do not extend beyond the lumbosacral junction or invade the small pelvic organs. Zang et al. (Zhang et al., 2009) published 10 posterior-only total *en bloc* resections of the sacrum and consider this technique to be a suitable and safe procedure for all sacral tumors. Sherman et al. (2012) described acceptable morbidity after the posterior-only approach for sacral tumor *en bloc* resection and identified obesity and operative time of more than 10 h as risk factors. McLoughlin et al. (2008) reported on total *en bloc* sacrectomy from posterior-only approach with sacral elevation to facilitate vascular control. By contrast, Roldan et al. (2014) described a low *en bloc* resection of the sacrum together with a rectal tumor from the anterior-only approach. We prefer combined anteroposterior surgical approaches for total and high *en bloc* sacrectomies, a posterior-only approach for middle and low, as well as lateral resections, and a combined posteroanterior approach for hemicorporectomy.

*En bloc* resection with a margin of healthy tissue is an essential criterion of a successful operative treatment of malignant sacral tumors. Bederman, Castiglione, Feghali, Fourney, Guo, Sciubba, Varga, Wei et al. (Bederman et al., 2014; Castiglione et al., 2020; Feghali et al., 2021; Fourney et al., 2005; Guo et al., 2013; Sciubba et al., 2009; Varga et al., 2014; Wei et al., 2019) published a robot-assisted resection within the anterior procedure in combination with a standard open posterior procedure. Farshad et al. (2021) described 3D printed osteotomy guide for a precise execution of low sacral resection.

Stability of the lumbo-sacroiliac complex is decisive for indication of an instrumented reconstruction. Instrumentation is not indicated in low resections with an intact sacroiliac joint (Bederman et al., 2014; Fourney et al., 2005; Štulík et al., 2020). On the contrary, in case of total sacral resection most authors favor iliolumbar stabilization allowing early rehabilitation (Fourney et al., 2005; Hugate et al., 2006; Morales-Codina and Martín-Benlloch, 2022; Murakami et al., 2002; Varga and Lazary, 2010). Bederman et al. (2014) distinguish between three types of reconstruction techniques: spinopelvic fixation (SPF), posterior pelvic ring fixation (PPRF) and anterior spinal column fixation (ASCF) and recommend their use in combination. Another group of authors avoid any instrumentation due to the risk of infection, loosening, prolonging of operative time etc. (Roldan et al., 2014; Wei et al., 2019). Wuisman et al. (2000) reported on 5 patients after total sacrectomy without instrumentation, where a caudal shift of the lumbar spine occurred, and a mass of scar tissue developed between the spine and the pelvis which provided a certain degree of stability with a satisfactory functional result after more than 8 weeks. In high resections between S1-S2, stability of the sacroiliac joints depends on the extent of resection. The complex is considered stable with preservation of at least 50 % of the surface of sacroiliac joints. This represents preservation of at least half of the S1 segment and stabilization is not necessary (Fourney et al., 2005; Hugate et al., 2006; Varga and Lazary, 2010; Zhu et al., 2012). Hugate et al. (2006) published a biomechanical study on incidence of S1 fractures following a high sacral resection on cadaveric material. With sacrectomy performed caudal to the S1 neural foramina, the average resection of the sacroiliac joints was 16 %, with sacrectomy performed cephalad to the S1 neural foramina it was 25 %. In lower resections, axial load caused Denis zone II fractures, in higher resections more often Denis zone III fractures, but bilateral. The authors recommend reconstruction and fixation in higher resections performed cephalad to the S1 neural foramina. Bergh et al. (2000), however, reported 33 % of stress fractures in their 18 patients after high sacral resections (S1-S2 and higher). A clinically long-term negative effect of the fracture was observed only in one patient. In our cohort of 14 patients after a high *en bloc* sacral resection (S1-S2) without fixation, we recorded 5 (35.7 %) fractures localized solely in Denis zone I or III (Denis et al., 1988). Clinically more severe complications were not observed.

Filling the space after total or high sacral resection and the subsequent wound suture with a high risk of wound healing problems are one of the main issues of operative treatment. Most authors prefer soft tissue reconstruction to avoid rectal prolapse and wound healing complications. The most frequently performed transfer technique uses vertical rectus abdominis musculocutaneous (VRAM) flap via the anterior approach or gluteus maximus adipomuscular (GM) flap from the posterior approach (Fourney et al., 2005; Varga et al., 2014) Kim et al. (2015) recommend VRAM flap technique for complex anteroposterior surgical approaches and GM flap technique for posterior approaches. 3D printed sacral endoprosthesis with or without a supplementing lumbopelvic fixation is an up-to-date option meeting both the above-mentioned requirements, i.e., stability of the complex and filling of the defect (Morales-Codina and Martín-Benlloch, 2022; Wei et al., 2019). Wei et al. (2019) published a cohort of 10 patients with a 3D printed sacral implant and report results in terms of stability of the complex and implant failure comparable to those provided by a combined technique of fixation. They recorded significantly better results only in comparison with posterior reconstruction and fixation. Morales-Codina and Martín-Benlloch (2022) evaluated various types of iliolumbar fixation alone or in combination with a 3D printed implant in their biomechanical study. With the use of a 3D printed implant, they found a marked decrease in stress values related to instrumentation and bone structures and assumed a reduced risk of instrumentation failure. Huang et al. (2019) also pointed out biomechanical benefits of a 3D printed implant and favor its use.

Complications after sacral resections are relatively frequent. In addition to the above-mentioned fractures, they include particularly wound healing problems or local recurrencies associated with non-radical procedures. Reynolds et al. recommend soft tissue reconstruction is performed after total sacrectomy allowing tension-free closure, and dead space elimination, thus reducing wound dehiscence and return to theater (Reynolds et al., 2016).

Potential functional impairment of the patient after *en bloc* sacral resection is an important part of the consenting process. Zoccali et al. (2016) focused in their review on neurological deficits, i.e., residual motor function and gait, sensitivity, bladder, bowel, and sexual function. In total sacrectomies, all functions are compromised to a certain degree, residual motor function depends on sparing L5 and S1 nerve roots. Essential for bladder and bowel function is preservation of at least one L3 nerve root. Unilateral resection is usually associated with a better clinical result. Fourney et al. (2005) report more severe impairment of sexual function in older patients.

## 5 Conclusion

Partial sacrectomy with *en bloc* tumor resection is an extensive surgical procedure which is often the only option which provides cure. Our experience shows that, in selected cases, instrumentation is not necessary even in case of a high *en bloc* partial sacrectomy (S1-S2 level) retaining the cranial part of the sacrum *in situ*. This creates suitable conditions for subsequent proton beam therapy. Stress fractures of the sacral stump occur in elderly patients with lower bone mineral density, or in younger patients with a higher bone mineral density who are more active when resuming their daily routine after the operation. Instrumentation is, in our view, primarily indicated in younger and more active patients, whereas in a majority of patients, even with lower bone mineral density, non-instrumented procedure results in sufficient stability with all levels of resection.

## Funding

This study was supported by Ministry of Health of Czech Republic - conceptual development of research organization, Motol University Hospital, Prague, Czech Republic (grant number: 00064203).

## Declaration of competing interest

The authors declare that they have no known competing financial interests or personal relationships that could have appeared to influence the work reported in this paper.

## References

[bib1] Bederman S.S., Shah K.N., Hassan J.M., Hoang B.H., Kiester P.D., Bhatia N.N. (2014). Surgical techniques for spinopelvic reconstruction following total sacrectomy: a systematic review. Eur. Spine J..

[bib2] Bergh P., Kindblom L.G., Gunterberg B., Remotti F., Ryd W., Meis-Kindblom J.M. (2000). Prognostic factors in chordoma of the sacrum and mobile spine: a study of 39 patients. Cancer.

[bib3] Boriani S., Weinstein J.N., Biagini R. (1997). Primary bone tumors of the spine. Terminology and surgical staging. Spine.

[bib4] Castiglione M., Conti C., Frondizi D., Cottini E., Cochetti G., Ciampini A., Cellini V., Mearini E. (2020). A combined one-staged robot-assisted sacral chordoma resection. World Neurosurg.

[bib5] Clarke M.J., Dasenbrock H., Bydon A., Sciubba D.M., McGirt M.J., Hsieh P.C., Yassari R., Gokaslan Z.L., Wolinsky J.P. (2012). Posterior-only approach for en bloc sacrectomy: clinical outcomes in 36 consecutive patients. Neurosurgery.

[bib6] Denis F., Davis S., Comfort T. (1988). Sacral fractures: an important problem. Retrospective analysis of 236 cases. Clin. Orthop. Relat. Res..

[bib7] Enneking W.F., Spanier S.S., Goodman M.A. (1980). A system for the surgical staging of musculoskeletal sarcoma. Clin. Orthop. Relat. Res..

[bib8] Farshad M., Selman F., Burkhard M.D., Müller D., Spirig J.M. (2021). Partial sacrectomy with patient-specific osteotomy guides. N Am. Spine Soc. J..

[bib9] Feghali J., Pennington Z., Hung B., Hersh A., Schilling A., Ehresman J., Srivastava S., Cottrill E., Lubelski D., Lo S.F., Sciubba D.M. (2021). Sacrectomy for sacral tumors: perioperative outcomes in a large-volume comprehensive cancer center. Spine J..

[bib10] Fourney D.R., Rhines L.D., Hentschel S.J., Skibber J.M., Wolinsky J.P., Weber K.L., Suki D., Gallia G.L., Garonzik I., Gokaslan Z.L. (2005). En bloc resection of primary sacral tumors: classification of surgical approaches and outcome. J. Neurosurg. Spine.

[bib11] Guo W., Tang X., Zang J., Ji T. (2013). One-stage total en bloc sacrectomy: a novel technique and report of 9 cases. Spine.

[bib12] Hsieh P.C., Xu R., Sciubba D.M., McGirt M.J., Nelson C., Witham T.F., Wolinksy J.P., Gokaslan Z.L. (2009). Long-term clinical outcomes following en bloc resections for sacral chordomas and chondrosarcomas: a series of twenty consecutive patients. Spine.

[bib13] Huang S., Ji T., Guo W. (2019). Biomechanical comparison of a 3D-printed sacrum prosthesis versus rod-screw systems for reconstruction after total sacrectomy: a finite element analysis. Clin. Biomech..

[bib14] Hugate RR Jr, Dickey I.D., Phimolsarnti R., Yaszemski M.J., Sim F.H. (2006). Mechanical effects of partial sacrectomy: when is reconstruction necessary?. Clin. Orthop. Relat. Res..

[bib15] Kim J.E., Pang J., Christensen J.M., Coon D., Zadnik P.L., Wolinsky J.P., Gokaslan Z.L., Bydon A., Sciubba D.M., Witham T., Redett R.J., Sacks J.M. (2015). Soft-tissue reconstruction after total en bloc sacrectomy. J. Neurosurg. Spine.

[bib16] McLoughlin G.S., Sciubba D.M., Suk I., Witham T., Bydon A., Gokaslan Z.L., Wolinsky J.P. (2008). En bloc total sacrectomy performed in a single stage through a posterior approach. Neurosurgery.

[bib17] Morales-Codina A.M., Martín-Benlloch J.A. (2022). Sacral prosthesis substitution as a system of spinopelvic reconstruction after total sacrectomy: assessment using the finite element method. Internet J. Spine Surg..

[bib18] Murakami H., Kawahara N., Tomita K., Sakamoto J., Oda J. (2002). Biomechanical evaluation of reconstructed lumbosacral spine after total sacrectomy. J. Orthop. Sci..

[bib19] Reynolds J.J., Khundkar R., Boriani S., Williams R., Rhines L.D., Kawahara N., Wolinsky J.P., Gokaslan Z.L., Varga P.P. (2016). Soft tissue and bone defect management in total sacrectomy for primary sacral tumors: a systematic review with expert recommendations. Spine.

[bib20] Roldan H., Perez-Orribo L.F., Plata-Bello J.M., Martin-Malagon A.I., Garcia-Marin V.M. (2014). Anterior-only Partial Sacrectomy for en bloc Resection of Locally Advanced Rectal Cancer. Glob. Spine J..

[bib22] Sciubba D.M., Petteys R.J., Garces-Ambrossi G.L., Noggle J.C., McGirt M.J., Wolinsky J.P., Witham T.F., Gokaslan Z.L. (2009). Diagnosis and management of sacral tumors. J. Neurosurg. Spine.

[bib23] Schreiber J.J., Anderson P.A., Hsu W.K. (2014). Use of computed tomography for assessing bone mineral density. Neurosurg. Focus.

[bib24] Sherman C.E., Rose P.S., Pierce L.L., Yaszemski M.J., Sim F.H. (2012). Prospective assessment of patient morbidity from prone sacral positioning. J. Neurosurg. Spine.

[bib25] Štulík J., Hoch J., Richtr P., Kříž J., Přikryl P., Kryl J. (2020). Hemikorporektomie jako nejvyšší stupeň en bloc resekce sakra [Hemicorporectomy as the Highest Grade of En Bloc Sacrectomy]. Acta Chir. Orthop. Traumatol. Cech..

[bib26] Varga P.P., Lazary A. (2010). Chordoma of the sacrum: "en bloc" high partial sacrectomy. Eur. Spine J..

[bib27] Varga P.P., Szövérfi Z., Lazary A. (2014). Surgical treatment of primary malignant tumors of the sacrum. Neurol. Res..

[bib28] Wei R., Guo W., Yang R., Tang X., Yang Y., Ji T., Liang H. (2019). Reconstruction of the pelvic ring after total *en bloc* sacrectomy using a 3D-printed sacral endoprosthesis with re-establishment of spinopelvic stability: a retrospective comparative study. Bone Joint Lett. J.

[bib29] Wuisman P., Lieshout O., Sugihara S., van Dijk M. (2000). Total sacrectomy and reconstruction: oncologic and functional outcome. Clin. Orthop. Relat. Res..

[bib30] Yu B.S., Zhuang X.M., Li Z.M., Zheng Z.M., Zhou Z.Y., Zou X.N., Lu W.W. (2010). Biomechanical effects of the extent of sacrectomy on the stability of lumbo-iliac reconstruction using iliac screw techniques: what level of sacrectomy requires the bilateral dual iliac screw technique?. Clin. Biomech..

[bib31] Zhang W., Liao X.J., Lou Z., Meng R.G., Yu E.D., Fu C.G., Yu D.H. (2009). [Transsacral resection for presacral tumors]. Zhonghua Wei Chang Wai Ke Za Zhi.

[bib32] Zhang H.Y., Thongtrangan I., Balabhadra R.S., Murovic J.A., Kim D.H. (2003). Surgical techniques for total sacrectomy and spinopelvic reconstruction. Neurosurg. Focus.

[bib33] Zhu R., Cheng L.M., Yu Y., Zander T., Chen B., Rohlmann A. (2012). Comparison of four reconstruction methods after total sacrectomy: a finite element study. Clin. Biomech..

[bib34] Zoccali C., Skoch J., Patel A.S., Walter C.M., Maykowski P., Baaj A.A. (2016). Residual neurological function after sacral root resection during en-bloc sacrectomy: a systematic review. Eur. Spine J..

