# Supplementary material for: Partial sacrectomy with en bloc tumor resection without instrumentation. What level is safe?
Source: Brain Spine. 2025 Mar 27;5:104246. doi: 10.1016/j.bas.2025.104246 (PMC11999580; doi:10.1016/j.bas.2025.104246)
Supplement: Multimedia component 1 [file mmc1.docx]

Kaplan Meier


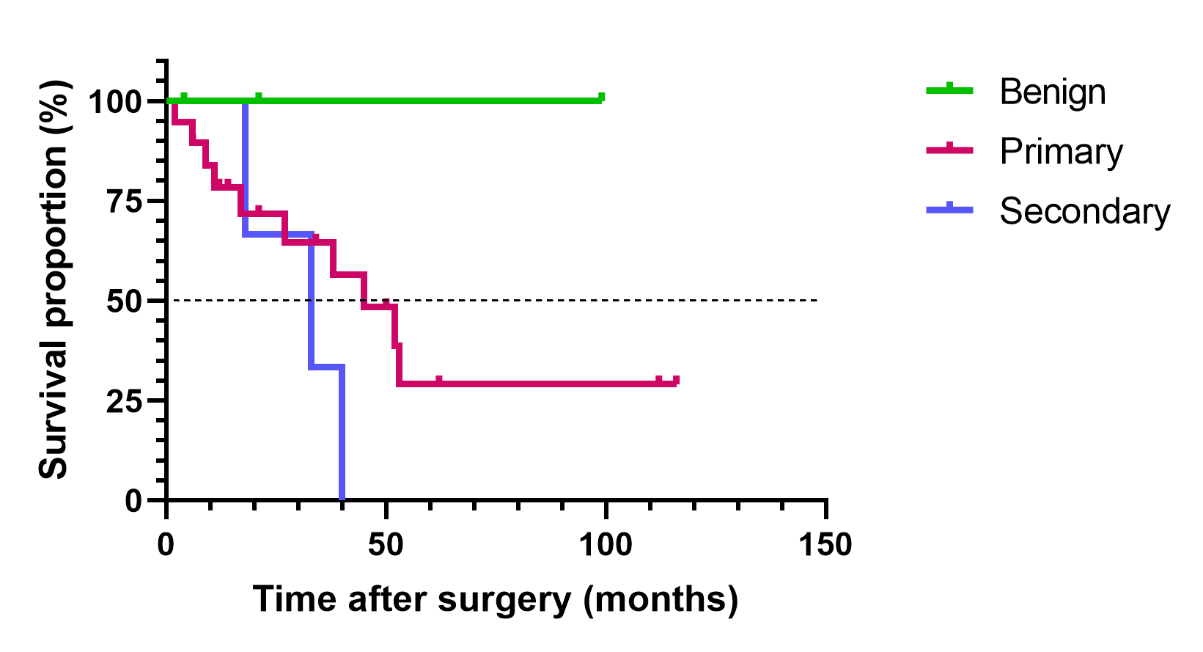


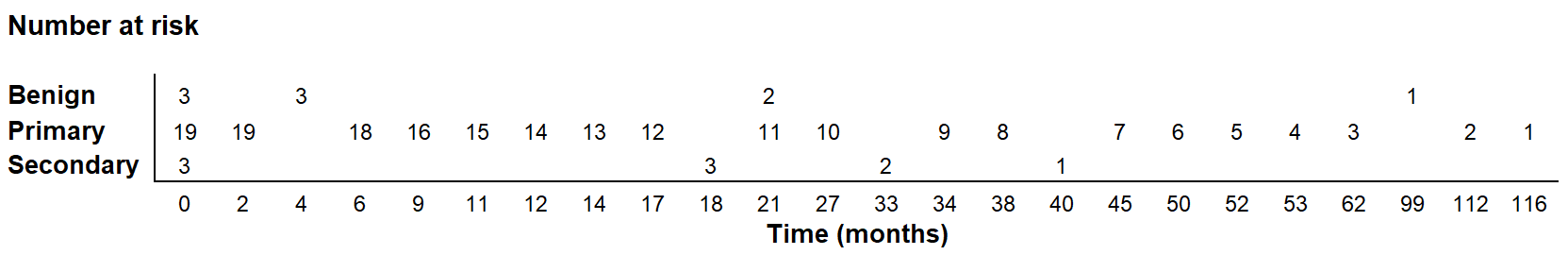


**Figure 1. Kaplan-Meier Survival Curves for Patients Undergoing Sacrectomy Stratified by Tumor Histology**
The Kaplan-Meier survival curves demonstrate survival outcomes for patients with benign (green line), primary (red line), and secondary (blue line) tumors. Patients with benign tumors had the most favorable survival outcomes, with no observed mortality throughout the follow-up period. Primary tumor patients experienced a steady decline in survival, while secondary tumor patients had the poorest prognosis, with a steep decline in survival within the first 30 months post-surgery. However, no statistically significant differences in survival were detected among the groups (p = 0.1891).

**Materials and Methods:**

Survival analysis was performed using the Kaplan-Meier method, and survival curves were generated for each tumor histology group. The Log-rank (Mantel-Cox) test was used to determine if there were significant differences in overall survival between the groups. A p-value of less than 0.05 was considered statistically significant. The statistical analysis was conducted using GraphPad Prism version 10.0.0 for Windows (GraphPad Software, Boston, Massachusetts USA).

**Results:**

The Kaplan-Meier survival curves (Figure 1) revealed distinct differences in survival among the three groups. Patients with benign tumors exhibited the most favorable outcomes, with a 100% survival rate maintained throughout the follow-up period. In contrast, patients with primary tumors experienced a gradual decline in survival over time, with the median survival falling below 50% by approximately 50 months post-surgery. The most pronounced decline was observed in the secondary tumor group, where a rapid decrease in survival was evident within the first 30 months. Despite these apparent differences, the statistical comparison of survival curves using the Log-rank (Mantel-Cox) test did not reach statistical significance (Chi-square = 3.331, df = 2, p = 0.1891). This suggests that, while the survival trends differ visually, these differences are not statistically significant, potentially due to the small sample sizes within the benign and secondary tumor groups.
